# Novel Thiazolidinedione Derivatives as Potential ZIKV Antiviral Inhibitors

**DOI:** 10.3390/microorganisms13091967

**Published:** 2025-08-22

**Authors:** Isabella Luiza Ralph de Oliveira, José Arion da Silva Moura, Patricia Recordon-Pinson, Floriane Lagadec, Michelle Melgarejo da Rosa, Sayonara Maria Calado Gonçalves, Douglas Carvalho Francisco Viana, Paulo André Teixeira de Moraes Gomes, Marina Galdino da Rocha Pitta, Moacyr Jesus Barreto de Melo Rêgo, Michelly Cristiny Pereira, Mathieu Métifiot, Marie-Line Andreola, Maira Galdino da Rocha Pitta

**Affiliations:** 1 Research Center for Therapeutic Innovation Suely Galdino, Federal University of Pernambuco, Recife 50670-901, Brazil; isabella.ralph@ufpe.br (I.L.R.d.O.); arion.moura@ufpe.br (J.A.d.S.M.); sayonaramcg@gmail.com (S.M.C.G.); douglas.viana@ufpe.br (D.C.F.V.); paulo.atmgomes@ufpe.br (P.A.T.d.M.G.); marinagaldinopitta@gmail.com (M.G.d.R.P.); moacyr.rego@ufpe.br (M.J.B.d.M.R.); michelly.pereira@ufpe.br (M.C.P.); maira.pitta@ufpe.br (M.G.d.R.P.); 2 Laboratory of Immunomodulation and New Therapeutic Approaches, Federal University of Pernambuco, Recife 50670-901, Brazil; 3 Laboratory of Design and Drug Synthesis, Federal University of Pernambuco, Recife 50670-901, Brazil; 4 UMR 5234, Microbiologie Fondamentale et Pathogénicité, Université de Bordeaux, CNRS, F-33076 Bordeaux, France; floriane.lagadec@u-bordeaux.fr (F.L.); mathieu.metifiot@u-bordeaux.fr (M.M.); marie-aline.andreola@u-bordeaux.fr (M.-L.A.); 5 UBL3 Platform, TBMCore, UAR 3427/US 005, Université de Bordeaux, CNRS, F-33076 Bordeaux, France

**Keywords:** antiviral, molecular docking, NS2B-NS3, NS5, thiazolidinedione, Zika virus

## Abstract

Zika virus (ZIKV) remains a pressing global health concern due to its association with congenital Zika syndrome and the current lack of approved antiviral therapies. In this study, we evaluated the antiviral activity of three novel thiazolidinedione derivatives, GQ-402, GQ-396, and ZKC-10, against ZIKV in vitro and investigated their potential molecular targets through in silico analysis. GQ-402 exhibited the highest antiviral potency, with an IC_50_ of 15.7 µM, while ZKC-10 achieved the most substantial reduction in viral RNA levels, as determined by RT-qPCR. Molecular docking studies identified GQ-396 as the top-ranked inhibitor of the NS2B-NS3 protease and NS5 RNA-dependent RNA polymerase, suggesting distinct mechanisms of action among the compounds. These findings highlight the therapeutic potential of thiazolidinedione derivatives and underscore the need for further investigation to develop effective treatments for ZIKV infection.

## 1. Introduction

Arboviruses, or arthropod-borne viruses, represent significant global public health challenges as they are transmitted by hematophagous arthropod vectors [1]. Among these vectors, mosquitoes of the genus *Aedes*, particularly *Aedes aegypti*, play a pivotal role in the urban transmission cycles of several mosquito-borne arboviruses, including chikungunya virus (CHIKV), dengue virus (DENV), and Zika virus (ZIKV) [2].

ZIKV was first isolated in 1947 from a rhesus monkey in Uganda’s Zika Forest during a yellow fever epidemic [3]. In Brazil, Zika virus (ZIKV) emerged as a significant public health concern between 2014 and 2015, triggering an epidemic that disproportionately affected pregnant women and was associated with a marked increase in cases of microcephaly [4]. As of 2017, Zika virus (ZIKV) had spread to 52 countries and territories throughout the Americas, leading to over 220,000 confirmed cases and an estimated 580,000 suspected cases [5]. Recognized by the World Health Organization (WHO) as a global public health threat, ZIKV infection has been associated with severe fetal malformations [6].

ZIKV belongs to the *Flavivirus* genus and is characterized by a single-stranded RNA genome enclosed within a lipid envelope. Its genome encodes a single polyprotein that is cleaved by both viral and host proteases, yielding functional structural proteins (capsid, prM, and E) as well as nonstructural proteins (NS1, NS2A, NS2B, NS3, NS4A, NS4B, and NS5 [7]. Due to their critical involvement in viral entry, maturation, and replication, these proteins constitute prominent targets for the development of antiviral therapeutics [8]. The NS2B protein is a vital cofactor for the NS3 protease domain, facilitating its proper folding and enzymatic activity. The NS2B/NS3 protease complex cleaves the viral polyprotein into essential proteins required for viral replication, such as the capsid protein and RNA-dependent RNA polymerase. Studies of inhibitors targeting the ZIKV NS2B/NS3 protease have provided important insights for the development of therapeutic interventions [9]. Another target, nonstructural protein 5 (NS5), has been identified as a particularly promising candidate for antiviral intervention. NS5 comprises an N-terminal methyltransferase (MTase) domain and a C-terminal RNA-dependent RNA polymerase (RdRp) domain, both of which are essential for viral RNA synthesis and transcription [10].

Heterocyclic compounds, particularly thiazolidine (TZD) derivatives, have attracted considerable interest in the development of novel antiviral agents due to their wide range of biological activities [11]. Thiazolidines, including thiazolidinedione derivatives characterized by a five-membered ring structure containing nitrogen and sulfur atoms, present versatile substitution opportunities at positions 2, 4, and 5, rendering them highly attractive to the pharmaceutical industry [12]. Thiazolidine derivatives have exhibited notable antiviral activity against a range of viruses, including chikungunya virus (CHIKV) [13], hepatitis B virus (HBV) [14], hepatitis C virus (HCV) [15], human immunodeficiency virus (HIV) [16], dengue virus (DENV) [17], and severe acute respiratory syndrome coronavirus 2 (SARS-CoV-2) [18]. Recent studies have suggested that TZD derivatives exhibit antiviral activity against flaviviruses, with mechanisms of action associated with the inhibition of the viral proteins NS2A/NS3 [19,20,21,22,23] or NS5 [24]. These findings highlight their potential as promising candidates for the development of effective antiviral therapeutics.

This study investigates the antiviral potential of thiazolidinedione derivatives against Zika virus (ZIKV). Ten derivatives were initially screened, with compounds such as GQ-402, GQ-396, and ZKC-10 exhibiting promising activity and subsequently selected for further analysis. The evaluation comprised an assessment of virus-induced cytopathic effect reduction, the quantification of viral RNA levels via reverse transcription quantitative polymerase chain reaction (RT-qPCR), and the determination of cell viability in the presence of the tested compounds. Additionally, molecular docking studies were performed to explore the potential interactions between the derivatives and the ZIKV NS2B-NS3 protease and NS5 polymerase in silico. These results highlight the potential of these compounds as novel candidates for ZIKV inhibition, contributing to ongoing efforts toward the development of effective antiviral therapies.

## 2. Materials and Methods

### 2.1. Chemistry

The progress of the reaction was monitored using thin-layer chromatography (TLC) and high-performance liquid chromatography (HPLC). The physical constants of the purified compounds were determined in capillary tubes using a Büchi M-565 melting point apparatus (Flawil, Switzerland). Structural characterization of the synthesized compounds was performed by nuclear magnetic resonance (NMR) spectroscopy, with spectra recorded on an Agilent 300 MHz spectrometer (Santa Clara, CA, USA); tetramethylsilane (TMS) was used as the internal standard, and chemical shifts are reported in δ (ppm) units. Mass spectrometric analyses were conducted using a Shimadzu GC-MS QP2010 Plus spectrometer (Kyoto, Japan) under electron impact (EI) ionization, as well as a MALDI-TOF instrument.

#### 2.1.1. Obtaining Compounds for Screening

**Figure 1:**
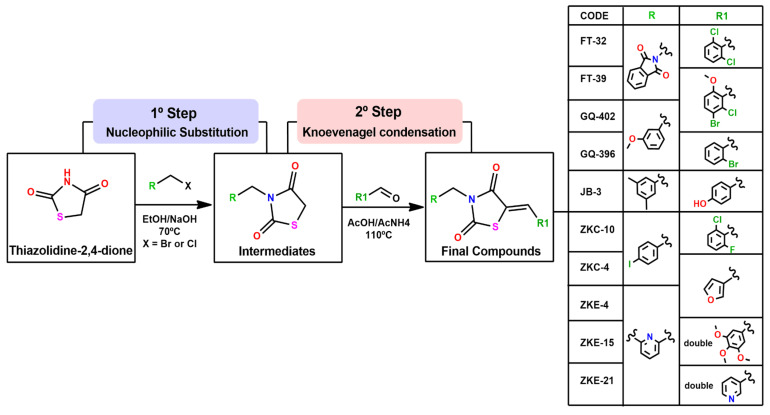
General synthetic procedure for thiazolidine derivatives. Step 1: Reaction of aromatic aldehydes with acetic acid and ammonium acetate at 110 °C for 4 h. Step 2: Reaction of intermediates with ketones in ethanol and a solution of NaOH or KOH at 65 °C for 4 h.

The compounds were designed and synthesized by the Laboratory of Pharmaceutical Synthesis and Planning, Federal University of Pernambuco, Brazil. Thiazolidine derivatives were synthesized, as illustrated in Figure 1. As a result, 10 compounds were obtained. Compounds FT-32 and FT-39 were previously produced by Oliveira et al. 2022 [25]. ZKC-4 was obtained by Gonçalves et al., 2024 [24]. Compounds ZKE-4, ZKE-15, and ZKE-21 were obtained by Moura et al. [26] and the compound JB-3 was obtained by Branco Junior et al. [27]. The synthesis procedures and spectroscopic data of the compounds selected in the screening (GQ-402, GQ-396, and ZKC-10) are presented below. Additional experimental details and data are available in the Supplementary Materials.

##### Procedure for Synthesis of 5-Benzylidene-3-(4-iodobenzyl)-thiazolidine-2,4-dione (ZKC-10)

The intermediate compound 3-(4-iodobenzyl)thiazolidine-2,4-dione (ZKC-1) was obtained by Gonçalves, 2024 [24]. The procedure for 1,3 thiazoles is described below: ZKC-1 (1.0 eq) and the corresponding aromatic aldehydes (1.0 eq) were mixed in acetic acid as the solvent for 30 min. Ammonium acetate (1.0 eq) was then added, and the reaction temperature was raised to 110 °C. After 3 to 4 h, the reaction mixtures were cooled and filtered to induce crystallization.

Yellow solid. C_17_H_10_ClFINO_2_S. m.p.: 88.1 °C. Yield: 52%. Purity: >99%. IR (KBr, cm^−1^): 780.24 (CBr), 898.87 (CF), 1690.68 (C=O), 1741.80 (CN). ^1^H NMR (300 MHz, DMSO-d6): 4.78 (s, 2H, CH2); 7.14 (d, 2H, J = 6.6 Hz, ArH 2,4 pos.); 7.40–7.59 (m, 3H, ArH 2,3,4 pos.); 7.73 (d, 2H, J = 6.6 Hz, ArH 2,4 pos.); 7.85 (s, 1H, CH). 13C NMR (300 MHz, DMSO-d6): δ = 44.46 (CH2), 94.01 (CI, Ar), 115.23 (CF, Ar), 115.52 (C4), 119.94 (C4, Ar), 124.89 (CH, Ar), 126.14 (CH, Ar), 126.19 (CH, Ar), 130.11 (CCl Ar), 134.97 (C4, Ar), 137.39 (CH, Ar), 157.25 (CH), 160.59 (CF, Ar), 164.50 (C=O), 166.57 (C=O); MS (m/z): [M + DMSO + H]^+^ 552.590 (3213 Intens), calculated 472.91 for [M + H]^+^.

##### General Procedure for Synthesis of 5-Benzylidene-3-(3-methoxybenzyl)-thiazolidine-2,4-dione (GQs)

The compound 3-(3-methoxybenzyl)thiazolidine-2,4-dione (1.0 eq) and the corresponding aromatic aldehydes (1.0 eq) were mixed in acetic acid as the solvent for 30 min. Ammonium acetate (2.0 eq) was then added, and the reaction temperature was raised to 110 °C. After 24 h for GQ-396 and 2 h for GQ-402, the reaction mixtures were cooled and filtered to induce crystallization. GQ-402 was further purified by recrystallization from ethanol.

##### 5-(2-Bromobenzylidene)-3-(3-methoxybenzyl)thiazolidine-2,4-dione (GQ-396)

Yellow solid. C_18_H_14_BrNO_3_S. m.p.: 102. Yield: 82%. Purity: >99%. IR (KBr, cm^−1^): 1262,46 (C-O); 1338,65 (C-N); 1682,96 (C=O); 1737,94 (C=O); ^1^H NMR (300 MHz, DMSO-d6): 3.71 (s, 3H, CH3); 4.8 (s, 2H, CH2); 6.9 (m, 1H ArH); 7.3 (t, 1H, ArH); 7.45 (t, 1H, ArH); 7.56 (m, 2H, ArH); 7.8 (d, 1H, ArH); 8.0 (s, 1H, CH). 13C NMR (300 MHz, DMSO-d6): δ = 44.711 (CH2); 55.041 (CH3); 113.069 (CH Ar); 113.590 (CH Ar); 119.628 (C=C); 124.916 (CH Ar); 125.269 (CBr Ar); 128.641 (CH Ar); 129.131 (CH Ar); 129.805 (CH Ar); 131.047 (CH Ar); 132.212 (C=C Ar); 133.607 (C=C Ar); 136.733 (CH); 159.340 (C-O Ar); 165.088 (C=O); 167.111 (C=O). MS m/z (%): 402.75 (11.77), calculated (402.98).

##### 5-(5-Bromo-2-methoxybenzylidene)-3-(3-methoxybenzyl)thiazolidine-2,4-dione (GQ-402)

Yellow solid. C_19_H_16_BrNO_4_S. m.p.: 123. Yield: 34%. Purity: >97%. IR (KBr, cm^−1^): 1249,93 (C-O); 1267,29 (C-O); 1328,05 (C-N); 1681,03 (C=O); 1738,90 (C=O); ^1^H NMR (300 MHz, DMSO-d6): 3.3 (s, 1H,); 3.62 (s, 3H, CH3); 3.82 (s, 3H, CH3); 4.77 (s, 2H, CH2); 6.85 (t, 3H, CH Ar); 7.1 (d, 1H, CH Ar); 7.25 (t, 1H, CH Ar); 7.5 (s, 1H, CH Ar); 7.6 (s, 1H, CH Ar); 7.95 (s, 1H, CH). 13C NMR (300 MHz, DMSO-d6): δ = 44.575 (CH2); 55.028 (CH3); 56.100 (CH3); 112.151 (CH Ar); 113.039 (CH Ar); 113.515 (CH Ar); 114.250 (CBr Ar); 119.553 (C=C); 123.048 (C=C Ar); 123.569 (CH Ar); 127.125 (CH Ar);129.776 (CH Ar); 131.140 (CH Ar); 134.758 (C=C Ar); 136.827 (CH); 157.012 (C-O); 159.326 (C-O); 165.319 (C=O); 167.112 (C=O).

### 2.2. Biology

#### 2.2.1. Viruses and Cell Lines

The African green monkey kidney epithelial cell line (Vero E6) was kindly provided by Vincent Pitard, UMR 5164—University of Bordeaux, France. Cells were maintained in Dulbecco’s Modified Eagle Medium (DMEM; Gibco, Grand Island, NY, USA), supplemented with 10% fetal calf serum (FCS) and gentamicin (50 μg/mL; G418, Gibco-BRL). Cultures were incubated at 37 °C in a humidified atmosphere containing 5% CO_2_. The Zika virus strain (ATCC^®^ VR-1843™) was generously provided by Dr. Vincent Calvez (Hôpital Pitié-Salpêtrière, Paris, France). All procedures involving infectious ZIKV were conducted in a Class III biosafety cabinet under biosafety level 3 (BSL-3) conditions at the UB’L3 facility (TransBioMed Core, University of Bordeaux, Bordeaux, France), in compliance with institutional biosafety regulations.

#### 2.2.2. Viral Production

The Zika virus (ZIKV) strain was propagated by infecting Vero E6 cells at a multiplicity of infection (MOI) of 0.1, followed by incubation at 37 °C in a humidified 5% CO_2_ atmosphere until cytopathic effects were evident, approximately 96 h post-infection. The culture supernatant was subsequently clarified by centrifugation (5 min at 1500 rpm), and aliquots were stored at −80 °C. Viral stocks were subjected to whole-genome sequencing using both Sanger and Oxford Nanopore technologies to verify the absence of culture-induced mutations. Viral titers were quantified by infecting 1 × 10^4^ Vero E6 cells in 96-well plates with serial dilutions in supplemented DMEM. Eight replicates were performed. Plates were incubated at 37 °C for 3 days and subsequently examined for cytopathic effects. The cytopathic effect was quantified using the CellTox™ Green Cytotoxicity Assay (Promega, Charbonnières-les-Bains, France) following the manufacturer’s instructions, and fluorescence was measured with a Victor Nivo reader (PerkinElmer, Waltham, MA, USA). The TCID_50_ per milliliter was calculated according to the Reed and Muench method. Viral titers, expressed as plaque-forming units (PFUs) per milliliter, were mathematically converted from the TCID_50_ values using the Poisson equation as follows: PFU/mL = −ln(0.5) × TCID_50_/mL.

#### 2.2.3. Cell Viability Assays

The cells were seeded in 96-well plates at a density of 1 × 10^4^ cells per well and incubated for 24 h at 37 °C in a 5% CO_2_ atmosphere. After this period, the compounds (or DMSO as a solvent control) were added at various concentrations (5 μM, 12.5 μM, 25 μM, 50 μM, and 100 μM), maintaining a final DMSO concentration of 0.5%. Following 72 h of treatment, cell viability was assessed using the CellTiter 96^®^ Aqueous One Solution Cell Proliferation Assay (G358A; Promega, Charbonnières-les-Bains, France) after a 2 h incubation at 37 °C. Absorbance was measured at 492 nm using an Apollo LB 911 ELISA Reader (Berthold Technologies GmbH & Co. KG, Bad Wildbad, Germany). Normalization of the data was performed by expressing the absorbance of each test compound as a percentage of the DMSO control ([test compound absorbance × 100]/DMSO control absorbance), with values normalized to 100%. Data represent the mean of two independent replicates performed in triplicate. Statistical analyses and figure preparation were conducted using Microsoft Excel and GraphPad Prism (version 8).

#### 2.2.4. Antiviral Measurement

Vero E6 cells were cultured in Dulbecco’s Modified Eagle Medium (DMEM) supplemented with 10% fetal calf serum and gentamicin (50 μg/mL). Cells were seeded in 96-well plates at a density of 1 × 10^4^ cells per well and incubated for 24 h at 37 °C in a 5% CO_2_ atmosphere. After this incubation period, the culture medium was removed, and the cells were infected with Zika virus (ZIKV) at a multiplicity of infection (MOI) of 0.1. Subsequently, the cells were treated with compounds at various concentrations (5 μM, 12.5 μM, 25 μM, 50 μM, and 100 μM) or with 0.5% DMSO as the solvent control. The cytopathic effect (CPE) after 72 h of treatment with the respective compounds was evaluated using the CellTox™ Green Cytotoxicity Assay (Promega). Luminescence signals were measured using a Victor Nivo reader (PerkinElmer) with excitation/emission settings of 530/30 nm. Data were derived from three independent replicates performed across two biological experiments. Percent CPE was calculated as [test compound)/(mortality) × 100. The 50% inhibitory concentration (IC_50_) was determined from the mean dose–response curve using non-linear regression analysis. Data were normalized to a maximum value of 100%.

#### 2.2.5. Viral RNA Quantification

Vero E6 cells cultured in DMEM supplemented with 10% fetal calf serum were seeded in 96-well plates at a density of 1 × 10^4^ cells per well and incubated for 24 h at 37 °C in a 5% CO_2_ atmosphere. After this incubation, compounds at varying concentrations (5 μM, 12.5 μM, 25 μM, 50 μM, and 100 μM) and 0.5% DMSO as the solvent control were added, followed by a 2 h incubation. Subsequently, the culture medium was removed, and the cells were infected with Zika virus (ZIKV) at a multiplicity of infection (MOI) of 0.1 in 100 µL for a 2 h adsorption period. After 2 h, the medium was removed, and compounds diluted in DMEM were added at concentrations of 5 μM, 12.5 μM, 25 μM, 50 μM, and 100 μM, followed by incubation for 24 and 48 h. At 24 and 48 h post-infection (hpi), supernatants were collected and stored at −80 °C for subsequent RNA extraction. Total RNA was isolated using the High Pure Viral RNA Kit (Roche, Basel, Switzerland) according to the manufacturer’s protocol. Viral RNA quantification was performed by reverse transcription quantitative polymerase chain reaction (RT-qPCR) using the GoTaq^®^ 1-Step RT-qPCR System kit (Promega).

Zika virus RNA was amplified using the forward primer ZFB1086 (5′-CCGCTGCCCAACACAAG-3′) and the reverse primer ZRB1162 (5′-CCACTAACGTTCTTTTGCAGACAT-3′), targeting a genomic region between the M and NS1 genes. The amplification protocol commenced with a reverse transcription step at 50 °C for 30 min, followed by an initial denaturation at 95 °C for 10 min. This was succeeded by 40 amplification cycles consisting of denaturation at 95 °C for 10 s, annealing at 60 °C for 10 s, and extension at 72 °C for 10 s. A melting curve analysis was performed by increasing the temperature at 0.5 °C per second from 60 °C to 95 °C. Viral RNA copy numbers were quantified by comparison to a standard curve generated from serial dilutions of ZIKV RNA molecules and are expressed as RNA copies per milliliter (copies/mL).

### 2.3. Molecular Docking of Ligands with Proteins

The compounds ZKC-10, GQ-396, and GQ-402 were initially constructed using ChemDraw Ultra 12.0 software. Subsequently, structural minimization was performed employing Avogadro software (https://avogadro.cc/) with the MMFF94s force field, utilizing the Steepest Descent algorithm configured with four steps per update. The protein structures targeted in this study, retrieved from the Protein Data Bank (PDB), include Zika virus NS5 methyltransferase (PDB ID: 5WXB) and NS2B-NS3 protease (PDB ID: 6KK6). Subsequently, molecular docking simulations were performed using GOLD 2022.3.0 software (https://www.ccdc.cam.ac.uk/solutions/software/gold/), employing the ChemPLP fitness function for docking scoring. The grid coordinates were centered on the co-crystallized ligand binding sites, with a radius of 10 Å for NS2B-NS3 and 15 Å for NS5 methyltransferase. Redocking validation yielded root-mean-square deviation (RMSD) values of 1.098 Å for NS2B-NS3 and 1.257 Å for NS5 MTase, indicating accurate reproduction of the binding poses. Intermolecular interactions were visualized using Discovery Studio Visualizer 2021 Client.

### 2.4. Statistical Analysis

The IC_50_ and R^2^ values were log_10_-transformed and calculated using a three-parameter dose–response inhibition model following the normalization of enzymatic activity data. Data analysis and figure preparation were performed using GraphPad Prism (version 8.0.1).

## 3. Results

### 3.1. Synthesis of the Compounds

The ten compounds evaluated in this study are thiazolidine-2,4-dione (TZD) derivatives, obtained from the molecular collection at the Laboratory of Drug Design and Synthesis, Federal University of Pernambuco, Brazil. The synthesis of TZD was carried out as previously described [25], via a cyclization reaction between thiourea and chloroacetic acid. The thiazolidine derivatives were synthesized through a two-step process, as illustrated in Figure 1. In the first step, intermediates were generated through a proposed nucleophilic substitution reaction. Structural characterization was performed by spectroscopic techniques, including proton nuclear magnetic resonance (^1^H NMR), which confirmed the presence of characteristic signals such as methylene (CH_2_) groups, thereby validating the success of the nucleophilic substitution.

In the second step, the final compounds were synthesized via a proposed Knoevenagel condensation reaction involving the intermediates, ammonium acetate, and aromatic aldehydes under elevated temperature conditions. Structural confirmation was achieved through comprehensive spectroscopic analyses, including ^1^H NMR and ^13^C nuclear magnetic resonance (NMR) spectroscopy, which exhibited characteristic signals consistent with the expected molecular frameworks. Additionally, infrared (IR) spectroscopy revealed the anticipated absorption bands, and mass spectrometry confirmed the expected molecular weights for each compound.

### 3.2. Screening of Ten Thiazolidinedione Derivatives Against Zika Infection in Vero E6 Cells

**Figure 2:**
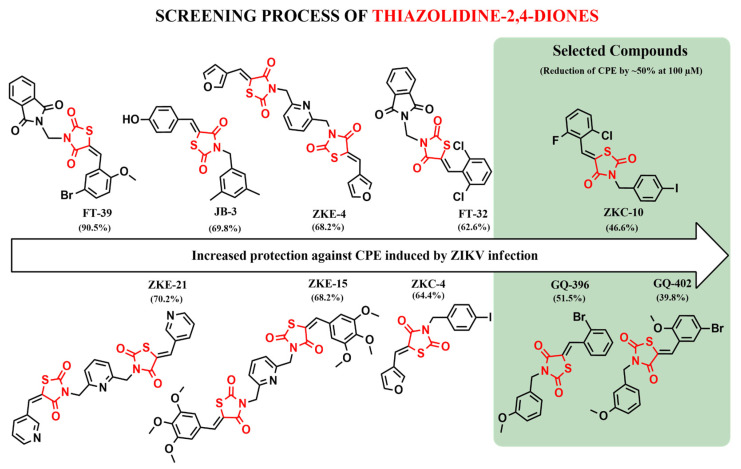
Screening of antiviral effect with 10 drugs for ZIKV at a 100 µM dose. Thiazolidinedione protects against cytopathic effects induced by ZIKV infection. Cytopathic effect (CPE) was observed when Vero E6 cells were infected by ZIKV and was determined by CellToXTM Green Cytotoxicity Assay luminescence method (100% mortality). The protection of the 10 molecules at 100 µM was evaluated by assessing cell mortality in ZIKV-infected Vero E6 cells. Individual values (% of mortality) represent the mean of the three replicates in one biological experiment.

In this study, Vero E6 cells were infected with ZIKV to perform the preliminary screening of synthetic heterocyclic compounds. A total of ten compounds were evaluated for their antiviral activity against ZIKV. Compounds FT-39, FT-32, JB-3, ZKC-4, ZKE-4, ZKE-15, and ZKE-21 were excluded due to their limited efficacy in inhibiting viral replication. Following this screening, three compounds that reduced cytopathic effects (CPEs) in infected cells by approximately 50% at a concentration of 100 µM were selected for further analysis (Figure 2).

**Figure 3:**
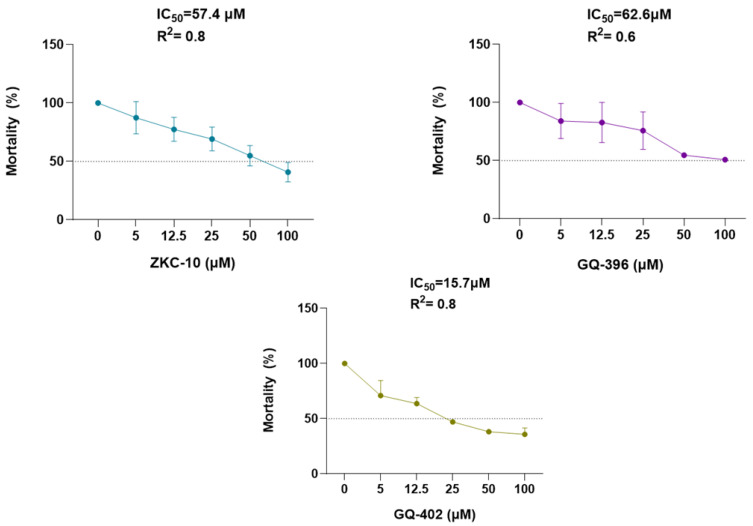
Dose–response curve analysis of the three compounds against ZIKV. Cytopathic effect (CPE) inhibition was measured using the CellTox™ Green Cytotoxicity Assay via luminescence detection. The IC_50_ and R^2^ values are indicated. Data points represent mean ± standard deviation (SD) from three independent replicates across two biological experiments. Values are plotted as line graphs with error bars depicting SD.

The antiviral activity of the three compounds, ZKC-10, GQ-396, and GQ-402, was assessed via dose–response analysis, focusing on their capacity to attenuate virus-induced cytopathic effects (CPEs) (Figure 3). Among these, GQ-402 demonstrated the most potent protective effect against ZIKV, followed by ZKC-10 and GQ-396. Specifically, GQ-402 inhibited CPEs by more than 50% at a concentration of 100 µM, with an IC_50_ value of 15.7 μM. In comparison, GQ-396 and ZKC-10 exhibited IC_50_ values of 62.6 μM and 57.4 μM, respectively.

### 3.3. Cytotoxicity and Antiviral Activity of Derivatives of Thiazolidinedione

**Figure 4:**
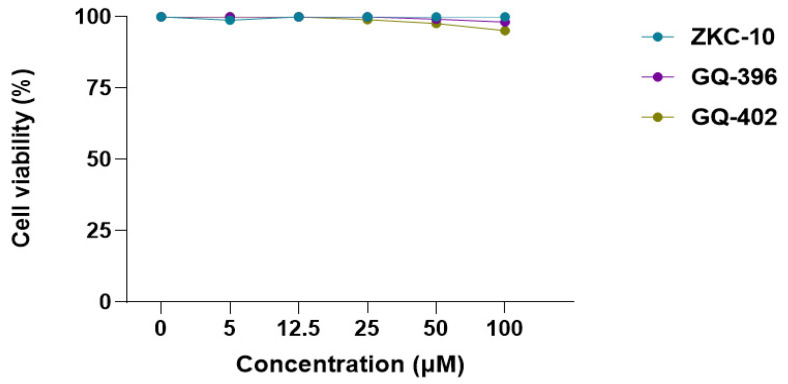
Viability of Vero E6 cells following 72 h treatment with ZKC-10, GQ-396, or GQ-402 at concentrations ranging from 5 µM to 100 µM, as assessed by the CellTiter 96^®^ Aqueous One Solution Cell Proliferation Assay. Data points represent the mean ± standard deviation (SD) of two replicates across three independent biological experiments. Cell viability is expressed as a percentage relative to the untreated control, which was set to 100%.

To confirm that the observed antiviral effects were not attributable to cytotoxicity, uninfected cells were treated with the compounds. The cytotoxicity of the three derivatives was assessed using a CellTiter assay. Vero E6 cell viability remained above 90% at all tested concentrations, indicating that none of the compounds exhibited cytotoxic effects (Figure 4). All three compounds exhibited more than 50% inhibition of CPEs without compromising cell viability at a concentration of 100 μM.

**Figure 5:**
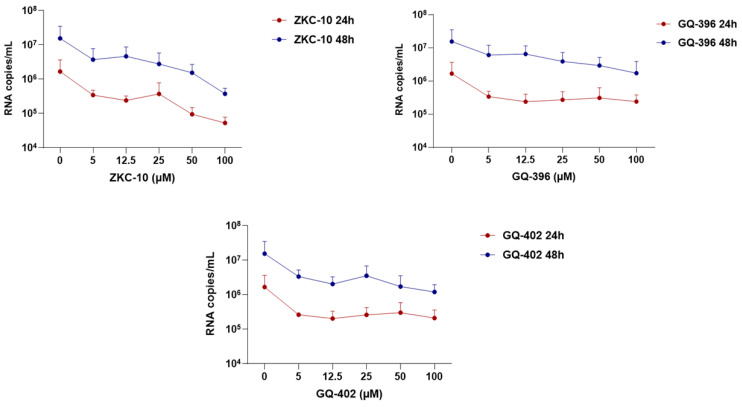
Effect of thiazolidinedione compounds on ZIKV RNA copy number in Vero E6 cells. Viral RNA levels were quantified by RT-qPCR at two distinct time points post-infection. Data points represent the mean of two technical replicates from three independent biological experiments.

All three compounds achieved greater than 50% inhibition of cytopathic effects (CPEs) at a concentration of 100 μM without adversely affecting cell viability. Furthermore, each compound effectively reduced viral RNA replication (Figure 5) without inducing cytotoxicity at this concentration. Notably, ZKC-10 exhibited a dose-dependent reduction in viral RNA levels relative to the other two compounds; however, this effect did not reach statistical significance at either time point evaluated (*p* > 0.05).

### 3.4. Molecular Docking of the Thiazolidinedione Derivatives

**Figure 6:**
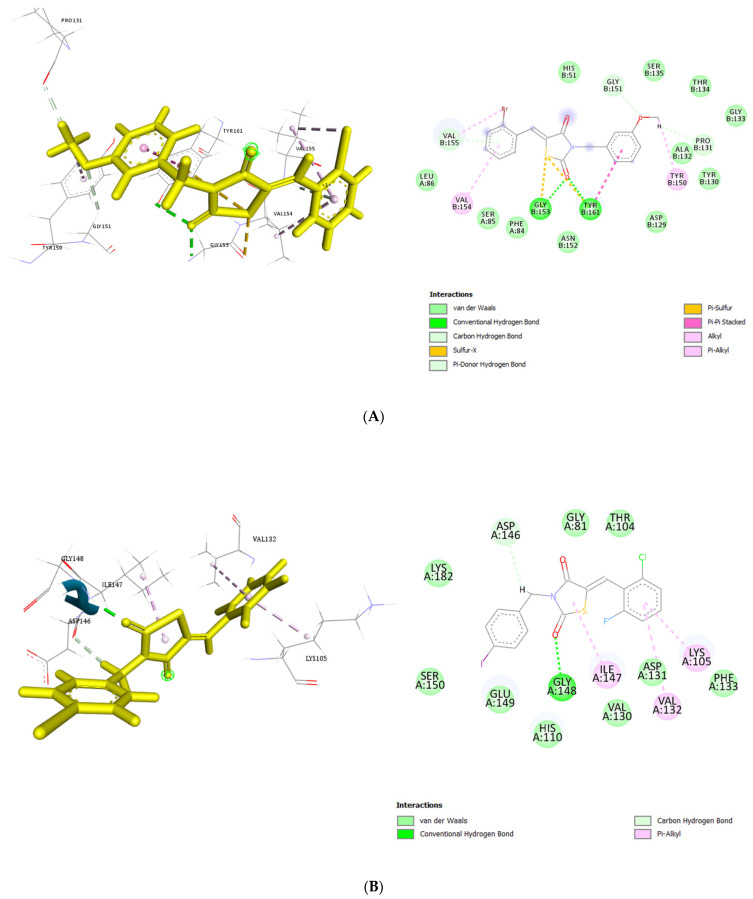
Crystal structures of Zika virus NS2B-NS3 protease and NS5 methyltransferase in complex with thiazolidinedione compounds. Molecular docking of compound GQ-396 within the active site of the NS2B-NS3 protease (**A**) and compound ZKC-10 within the active site of the NS5 methyltransferase (**B**).

Interaction scores between thiazolidinedione derivatives and Zika virus proteins were computed to evaluate binding affinities. The derivatives demonstrated notable affinity for the NS2B-NS3 protease and NS5 methyltransferase (MTase) targets. Specifically, the interaction scores calculated for NS2B-NS3 were 56.28 for GQ-402, 56.68 for GQ-396, and 55.27 for ZKC-10. Positive scores obtained using the GOLD software are indicative of favorable ligand–protein interactions [28]. Consequently, molecular docking results identified GQ-396 as possessing the highest predicted affinity for NS2B-NS3 (Figure 6A). The docking analyses were conducted using the Z isomeric conformations of the compounds, which, according to the literature, are generally more thermodynamically stable than their corresponding E isomers [29,30].

In this study, the first compound, GQ-402, formed conventional hydrogen bonds with Gly153 and Tyr161 and additional hydrogen bonds with Gly151 and Asp129. It also exhibited hydrophobic interactions, including π-π stacking and π-π T-shaped interactions with Tyr161, as well as alkyl interactions with Val154 and π-alkyl interactions involving Tyr161 and Val155.

The second compound, GQ-396, formed conventional hydrogen bonds with Gly153 and Tyr161, as well as sulfur interactions with Gly153, accompanied by π-π stacking with Tyr161. Additional interactions included hydrogen bonding with Gly151 and Pro131 and π-donor hydrogen bonding with Val155. Hydrophobic interactions comprised π-alkyl contacts with Tyr150, Val154, and Val155, with Val155 also exhibiting alkyl interactions.

The third compound, ZKC-10, formed a single hydrogen bond with Tyr161 and engaged in halogen bonding interactions with Asp129 and Tyr130 mediated by fluorine. Additionally, it exhibited hydrophobic interactions, including π-sigma interactions with Ala132 and π-π stacking with Tyr161, as well as alkyl interactions, including Leu86, Val154, and Val155.

The three compounds, ZKC-10, GQ-396, and GQ-402, exhibited hydrophobic interactions, π-π stacking, and hydrogen bonding interactions with Tyr161, a residue of critical importance. Notably, ZKC-10 demonstrated a hydrophobic π-sigma interaction with Ala132. The NS5 MTase protein, a key target in this study for antiviral drug discovery, plays an essential role in viral RNA cap methylation and replication [31]. Docking analyses of GQ-402, GQ-396, and ZKC-10 with NS5 MTase yielded binding scores of 58.99, 59.33, and 65.82, respectively. Among these, ZKC-10 exhibited the highest predicted affinity for NS5 (Figure 6), indicating a strong binding potential.

Key residues such as Phe133, Ser56, Gly81, Gly85, Trp87, Thr104, His110, Glu111, Asp131, and Glu146 are critical for the functional activity of NS5 MTase [32]. Specifically, GQ-402 exhibited significant interactions with Lys105, Gly148, and Ser150 through conventional hydrogen bonds, in addition to hydrophobic interactions, including alkyl and π-alkyl contacts with Gly148, Tyr103, and Val130.

GQ-396 formed conventional hydrogen bonds with Gly148 and additional hydrogen bonds with Asp146. It also established hydrophobic interactions, including π-δ contacts with Lys105 and Ile147, as well as π-π T-shaped interactions with Phe133. Furthermore, Val132 and Ile147 participated in π-alkyl interactions. Similarly, ZKC-10 engaged in conventional hydrogen bonding with Gly148 and Asp146 and exhibited π-alkyl interactions with Ile147, Lys105, and Val132. Collectively, the molecular docking analyses revealed significant interactions between the thiazolidinedione derivatives and key ZIKV targets.

## 4. Discussion

**Figure 7:**
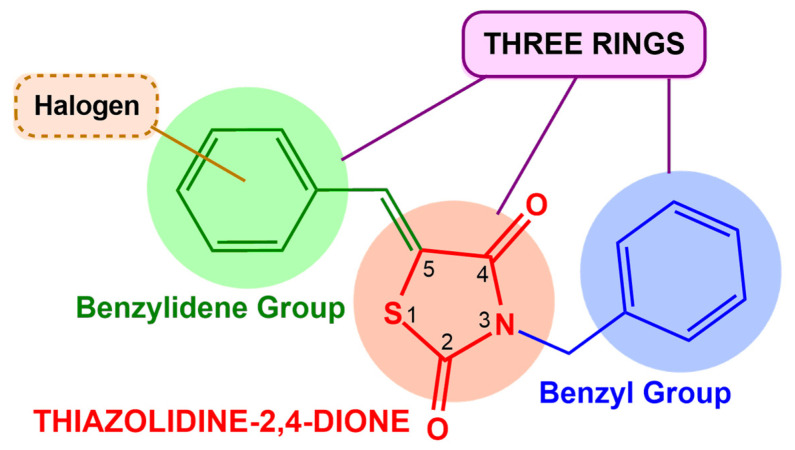
General structure of the most active compounds (ZKC-10, GQ-396, and GQ-402).

All evaluated molecules contain a thiazolidine-2,4-dione core substituted at positions 3 and 5. Among them, the three selected compounds, ZKC-10, GQ-396, and GQ-402, share several structural features. Each molecule comprises three interconnected rings: a central thiazolidine-2,4-dione ring, a benzyl group at position 3, and a benzylidene group at position 5, the latter bearing a halogen substituent. The presence of OCH_3_ or F substituents at the C-2 position of the benzene ring has been associated with enhanced antiviral activity against viruses such as HCV and HIV [33,34]. These shared characteristics may indicate a potential contribution to the antiviral mechanism of action against ZIK (Figure 7).

A study conducted in Vero cells assessed the 50% effective concentration (EC_50_) of antiviral activity using a cytopathic effect (CPE) reduction assay with thiazolidinedione (TZD) derivatives, reporting an EC_50_ value of 13 µM for ribavirin [35]. The data obtained in our study indicate that the antiviral activity of GQ-402 is comparable to that of ribavirin.

Another study evaluated the antiviral activity of an SRO-91 carboxamide derivative against ZIKV in Vero and Huh 7.5 cell lines, reporting IC_50_ values of 37.3 μM and 33.2 μM, respectively. In comparison, ribavirin exhibited IC_50_ values of 6.9 μM in Huh 7.5 cells and 29.9 μM in Vero cells. In our study, GQ-402 demonstrated antiviral activity against ZIKV in Vero cells comparable to that of ribavirin [36].

It is worth mentioning that ribavirin is a nucleoside analog structurally related to guanosine, acting as an antiviral agent through incorporation into the viral genetic material and induction of lethal mutagenesis, in addition to inhibiting viral enzymes such as RNA-dependent RNA polymerase (RdRp) [37]. However, nucleoside analogs often have limitations, including cellular toxicity and low selectivity, since they can interfere with host cellular processes [38]. In contrast, the compounds selected in this work are non-nucleoside inhibitors (NNIs). NNIs act on allosteric sites or other specific regions of viral proteins, such as NS2B/NS3 protease or NS5 polymerase, without the need for incorporation into the viral genome [39]. This approach tends to offer greater selectivity and lower toxicity, in addition to reducing the risk of side effects associated with interference with host cellular processes, which makes NNIs a promising and potentially safer alternative in the development of antivirals against ZIKV [40].

The molecular structure of GQ-396 features a bromine atom at position two of the aromatic ring, which is linked to the thiazolidinedione moiety at position five. In contrast, GQ-402 exhibits a similar structural framework but contains a methoxy group at position two and a bromine atom at position five of the thiazolidinedione ring. ZKC-10, however, belongs to a distinct series; its aromatic ring bears a 4-iodobenzyl substituent at position three of the thiazolidinedione core, along with ortho-fluoro (F) and ortho-chloro (Cl) substituents on the aryl group at position five. Previous studies have demonstrated that the incorporation of bromine atoms into chitosan derivatives enhances antiviral activity against the Newcastle disease virus [41]. Similarly, studies on oxalamide derivatives have demonstrated enhanced antiviral activity against human immunodeficiency virus type 1 (HIV-1) when substitutions such as 4-Cl and 4-CH_3_ groups were present, particularly in combination with 3-F substitutions on -CH_2_OH and -CH_2_CH_2_OH moieties [42].

Furthermore, computational approaches such as molecular docking facilitate the investigation of molecular mechanisms of action and support the identification of potential therapeutic candidates for various diseases [43]. Molecular docking is an in silico technique used to evaluate binding interactions between protein–ligand complexes, enabling the ranking of compounds based on predicted binding affinities or interaction scores [44].

Docking score analysis indicated that GQ-396 exhibited the strongest predicted interaction with the NS2B-NS3 complex, whereas ZKC-10 showed the lowest binding affinity. In vitro studies have confirmed that a flexible segment of the C-terminal region of NS2B adopts a more rigid conformation upon complex formation with NS3, a structural change essential for protease activity [45]. The hydrophilic central region of NS2B functions as a cofactor for NS3, playing a critical role in facilitating viral replication [46]. The NS3 protein, which includes the catalytic triad composed of Ser135, His51, and Asp75, forms the NS2B/NS3 protease complex [47], a structure essential for the cleavage of viral polyproteins. Targeting the NS2B-NS3 complex represents a promising strategy in the development of antiviral therapeutics [48]. The structural integrity of the NS2B protein, particularly its central β-barrel domain, contributes to the stabilization of NS3 [49].

Recent in silico studies have evaluated cinnamic acid derivatives against DENV-2 NS2B-NS3, revealing π-π stacking interactions with the Tyr161 residues [50]. Similarly, our thiazolidinedione derivatives demonstrated hydrophobic interactions with Tyr161, consistent with their antiviral activity observed in CPE inhibition assays. Notably, GQ-402, the most active compound with a docking score of 56.28, exhibited interactions predominantly within the NS2B subunit, including hydrogen bonds with Asp129, π-π stacking with Tyr161, and π-alkyl interactions with Val155.

A molecular dynamics (MD) study revealed strong interactions within the C-terminal region of the NS2B and NS3 proteins, highlighting the stability conferred by residues such as Asp75 and Asn152 interacting with Gly153, as well as Tyr161 interacting with P3, which are critical for inhibition. The NS2B protease cofactor facilitates interactions with the S2 pocket and inhibitors, playing an essential role in NS3 activation [51].

Most inhibitor derivatives interacted with the enzyme through hydrophobic interactions and hydrogen bonding, with residues Val154, Val155, and Tyr161 commonly involved. In a redocking analysis, hydrophobic contacts with Ala132 and π-π stacking interactions with Tyr161 were notably prominent, paralleling the interaction profile observed for the bioflavonoid rutin [52].

The docking results presented herein are based on the Z isomeric conformations, which are generally more thermodynamically stable than their corresponding E configurations [29,30]. Understanding the interactions of these derivatives with the NS2B-NS3 protease complex is critical, as this complex plays an essential role in the replication of flaviviruses [53]. Our findings are consistent with previous studies [54] investigating NS2B-NS3 interactions in related viruses such as DENV-2 and DENV-3, which identified key residues including Asp75, Asn152, and Tyr161.

## 5. Conclusions

The compounds GQ-402, GQ-396, and ZKC-10 exhibited antiviral activity against Zika virus (ZIKV), with ZKC-10 demonstrating the most potent inhibitory effect. Among the compounds evaluated, ZKC-10 emerges as a particularly promising candidate for further development as an antiviral agent against ZIKV, thereby warranting comprehensive studies to elucidate its mechanism of action and evaluate its potential clinical applications. This investigation utilized both in vitro assays and in silico modeling to assess the antiviral efficacy of three thiazolidine derivatives. Molecular docking analysis indicated that GQ-396 exhibited a significant binding affinity toward the NS2B-NS3 protease, as reflected by a docking score of 56.68, comparable to the affinities observed for the other compounds tested.

Despite exhibiting a significant molecular interaction, GQ-396 demonstrated the least pronounced reduction in cytopathic effect (CPE) relative to the other two compounds. In contrast, GQ-402 achieved a 39.8% reduction in CPE at a concentration of 100 µM, suggesting its potential efficacy in modulating ZIKV entry into host cells. Notably, in silico analysis revealed that ZKC-10 exhibited the strongest molecular interaction with the NS5 protein, indicating a considerable potential to inhibit ZIKV replication.

Although all three compounds reduced ZIKV viral load, ZKC-10 exhibited the most pronounced effect. Overall, the findings of this study highlight the antiviral potential of thiazolidine derivatives, with ZKC-10 emerging as a particularly promising candidate for the development of anti-ZIKV therapies. Nevertheless, further investigations are required to elucidate the precise mechanisms of action of thiazolidinedione derivatives in the context of ZIKV infection.

## Data Availability

The original contributions presented in this study are included in the article. Further inquiries can be directed to the corresponding author.

## Supplementary Materials

The following supporting information can be downloaded at: https://www.mdpi.com/article/10.3390/microorganisms13091967/s1.
