# Supplementary material for: Novel Thiazolidinedione Derivatives as Potential ZIKV Antiviral Inhibitors
Source: Microorganisms. 2025 Aug 22;13(9):1967. doi: 10.3390/microorganisms13091967 (PMC12471916; doi:10.3390/microorganisms13091967)
Supplement: Supplementary file 1 [file microorganisms-13-01967-s001.zip › microorganisms-3724828-supplementary.pdf]

ZKC-10

Chromatogram

ZKC-10 C:\LabSolutions\Data\ZKC-10.lcd

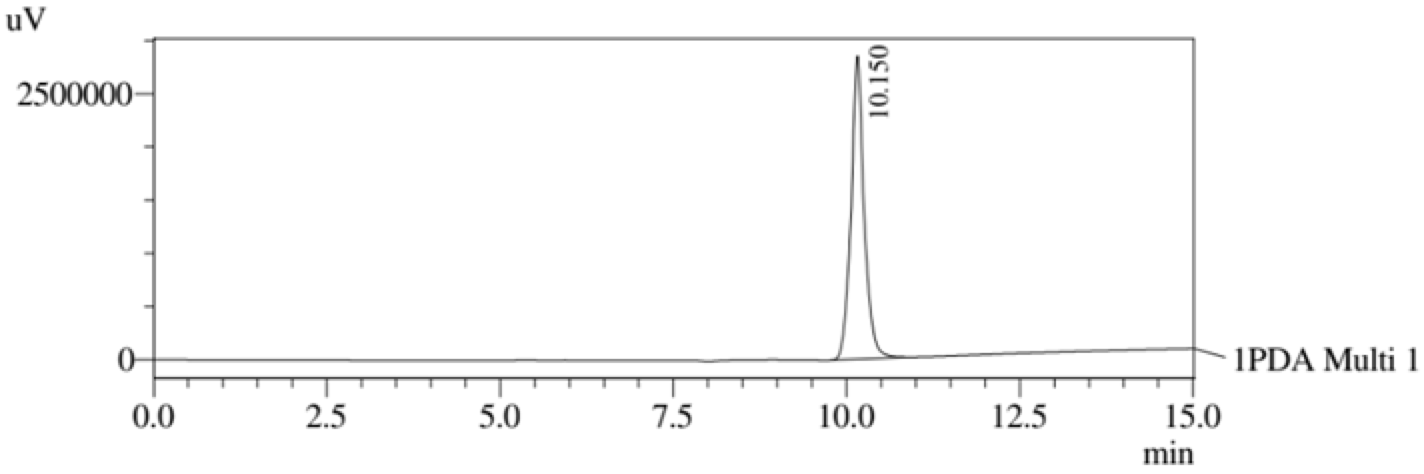

1 PDA Multi 1 / 205nm 4nm

<UV Library Filename>

<Search Parameters>

Search WL Range	: 190nm - 800nm
Max # of Hits	: 1

<Target Spectrum>

ID#	: 1
Retention Time	: 10.150
Compound Name	: ZKC-10
Spectrum Operation	: None

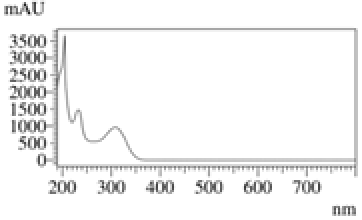

PeakTable

PDA Ch1 205nm 4nm

Peak#	Ret. Time	Area	Height	Area %	Height %
1	10.150	37518157	2849266	100.000	100.000
Total		37518157	2849266	100.000	100.000

ZKC-10

1H NMR

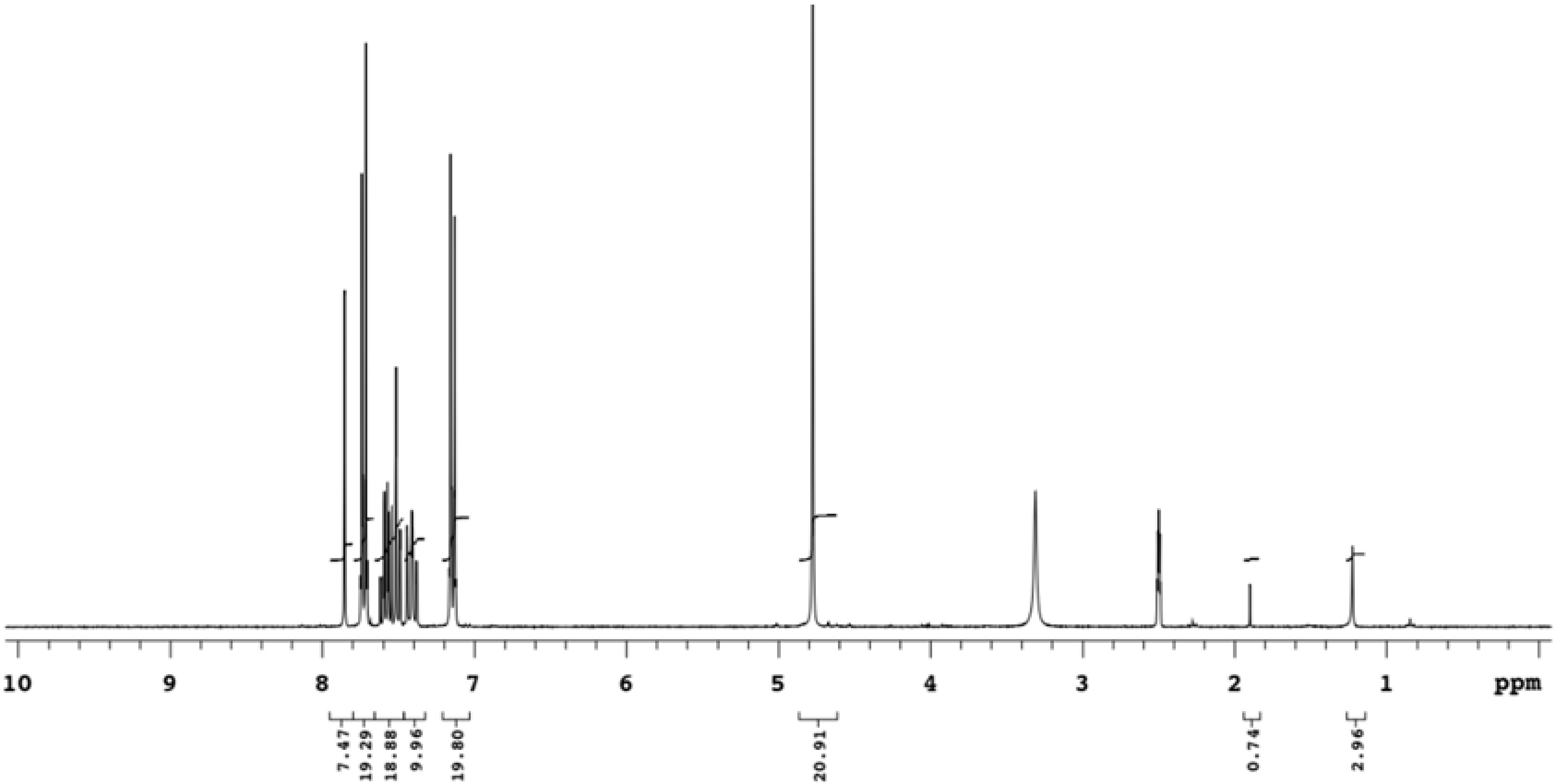

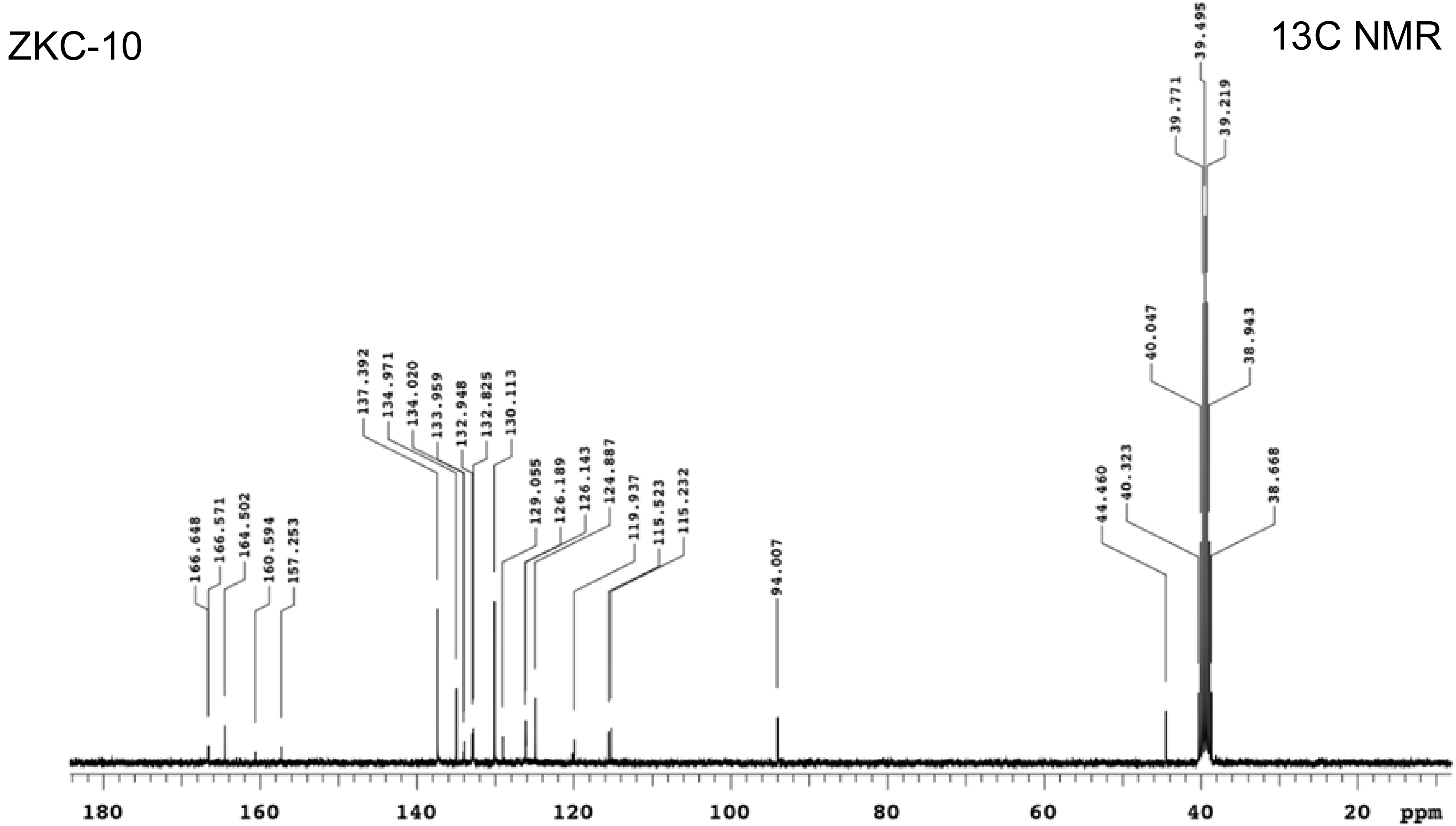
ZKC-10
13C NMR
166.648
166.571
164.502
160.594
157.253
137.392
134.971
134.020
133.959
132.948
132.825
130.113
129.055
126.189
126.143
124.887
119.937
115.523
115.232
94.007
44.460
40.323
40.047
39.771
39.495
39.219
38.943
38.668
180
160
140
120
100
80
60
40
20
ppm

IV

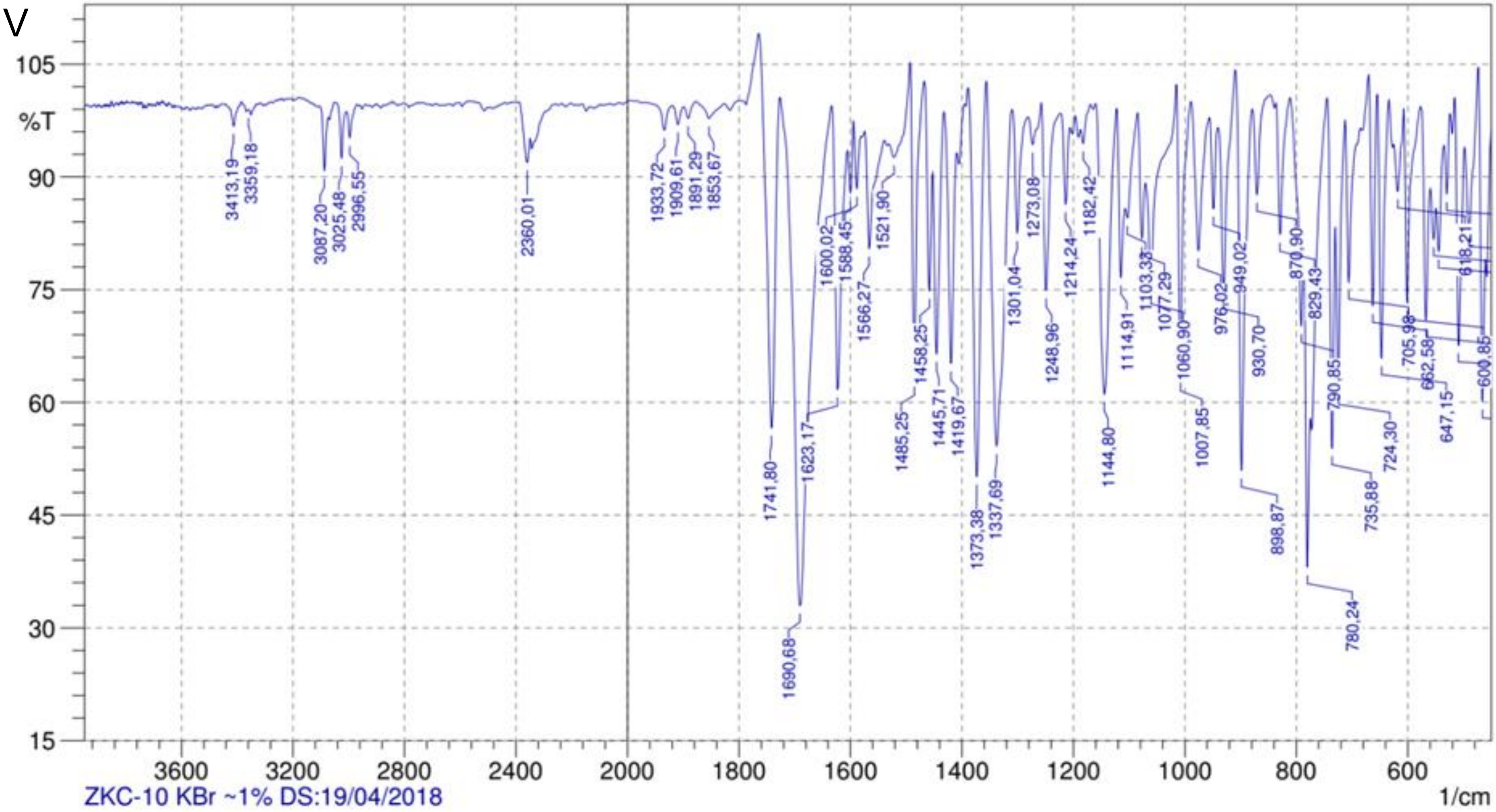
%T
105
90
75
60
45
30
15
3600
3200
2800
2400
2000
1800
1600
1400
1200
1000
800
600
1/cm
3413,19
3359,18
3087,20
3025,48
2996,55
2360,01
1933,72
1909,61
1891,29
1853,67
1741,80
1690,68
1623,17
1600,02
1588,45
1566,27
1521,90
1485,25
1458,25
1445,71
1419,67
1373,38
1337,69
1301,04
1273,08
1248,96
1214,24
1182,42
1144,80
1114,91
1103,33
1077,29
1060,90
1007,85
976,02
949,02
930,70
898,87
870,90
829,43
790,85
780,24
735,88
724,30
705,98
662,58
647,15
618,21
600,85
ZKC-10 KBr ~1% DS:19/04/2018

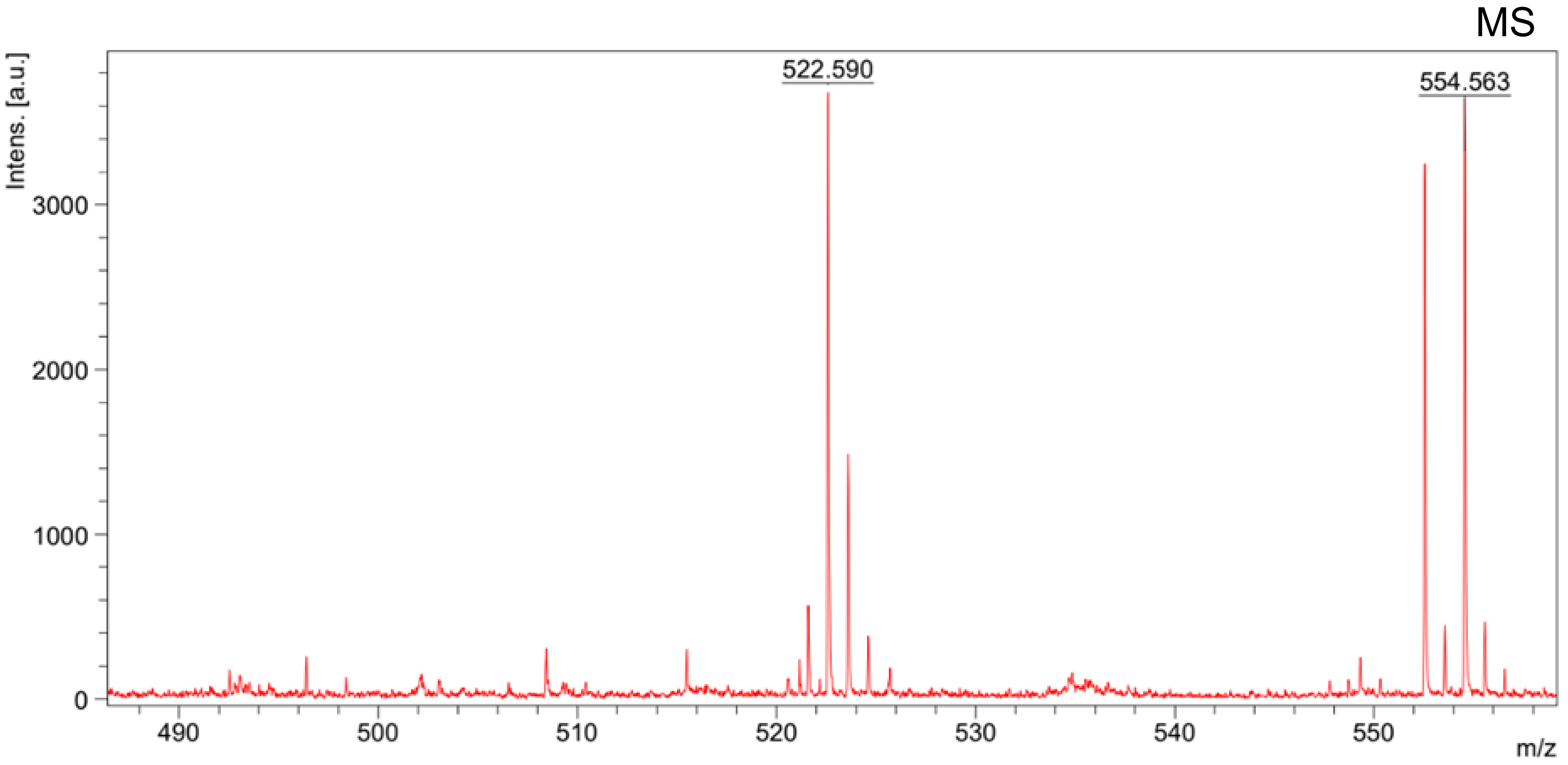
MS
522.590
554.563
Intens. [a.u.]
3000
2000
1000
0
490
500
510
520
530
540
550
m/z

GQ-402

Chromatogram
GQ-402 C:\LabSolutions\Data\GQ-402.lcd

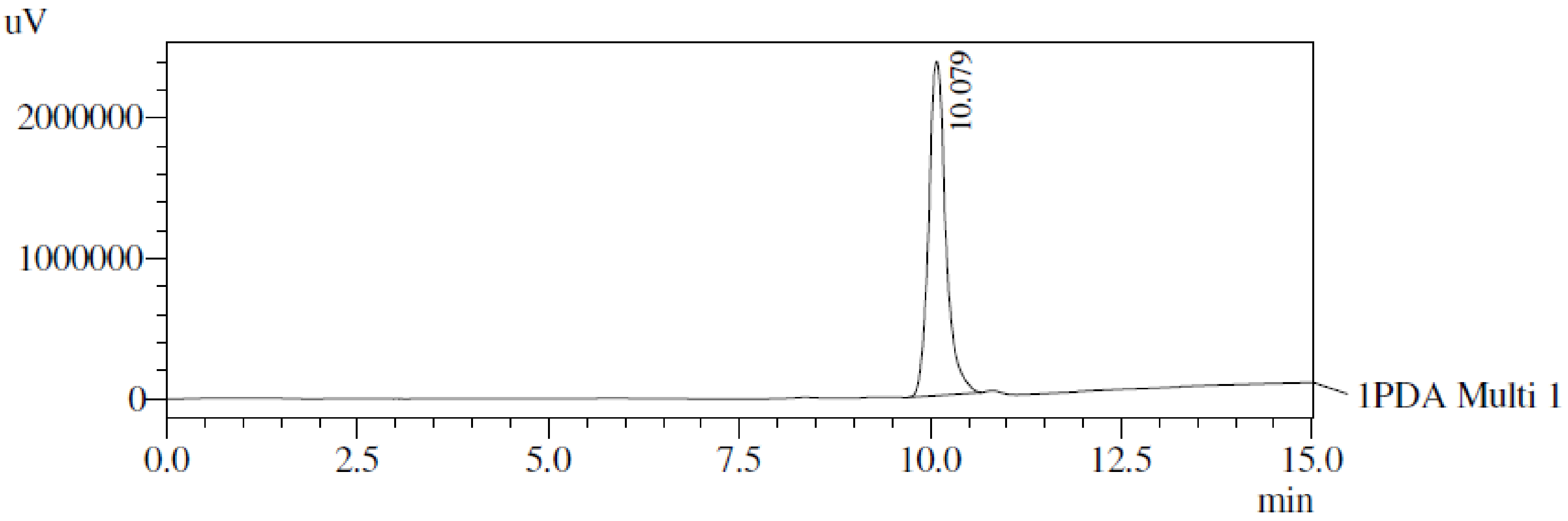

1 PDA Multi 1 / 205nm 4nm

<UV Library Filename>

<Search Parameters>
Search WL Range : 190nm - 800nm
Max # of Hits : 1

<Target Spectrum>
ID# : 1
Retention Time : 10.079
Compound Name : GQ-402
Spectrum Operation : None

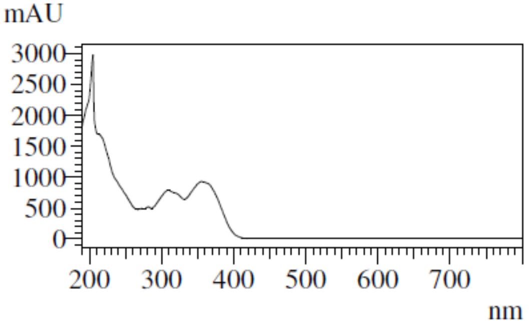

PeakTable

PDA Ch1 205nm 4nm

Peak#	Ret. Time	Area	Height	Area %	Height %
1	10.079	37036265	2378824	100.000	100.000
Total		37036265	2378824	100.000	100.000

GQ-402

1H NMR

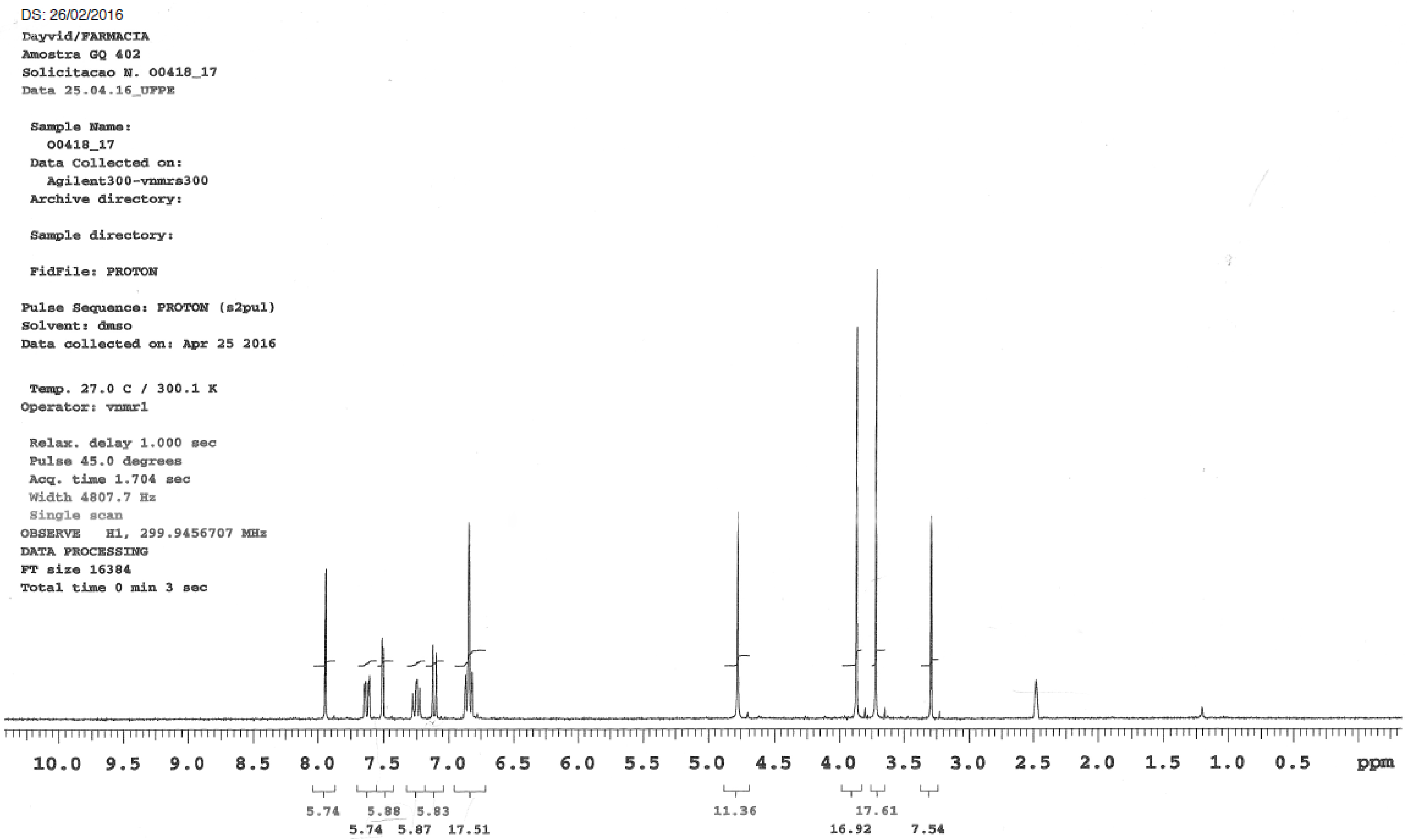

DS: 26/02/2016
Dayvid/FARMACIA
Amostra GQ 402
Solicitacao N. 00418_17
Data 25.04.16_UFPE
Sample Name:
00418_17
Data Collected on:
Agilent300-vnmrs300
Archive directory:
Sample directory:
FidFile: PROTON
Pulse Sequence: PROTON (s2pul)
Solvent: dmso
Data collected on: Apr 25 2016
Temp. 27.0 C / 300.1 K
Operator: vnmr1
Relax. delay 1.000 sec
Pulse 45.0 degrees
Acq. time 1.704 sec
Width 4807.7 Hz
Single scan
OBSERVE H1, 299.9456707 MHz
DATA PROCESSING
FT size 16384
Total time 0 min 3 sec
10.0 9.5 9.0 8.5 8.0 7.5 7.0 6.5 6.0 5.5 5.0 4.5 4.0 3.5 3.0 2.5 2.0 1.5 1.0 0.5 ppm
5.74 5.88 5.83 11.36 17.61
5.74 5.87 17.51 16.92 7.54

GQ-402

13C NMR

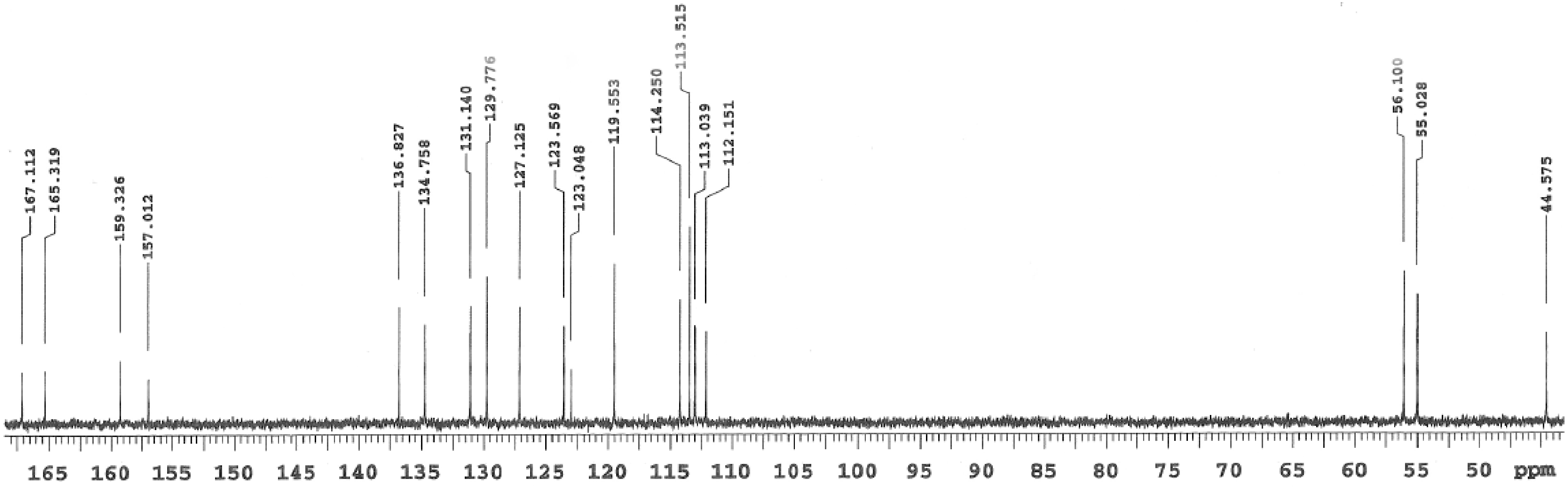

IV

SHIMADZU

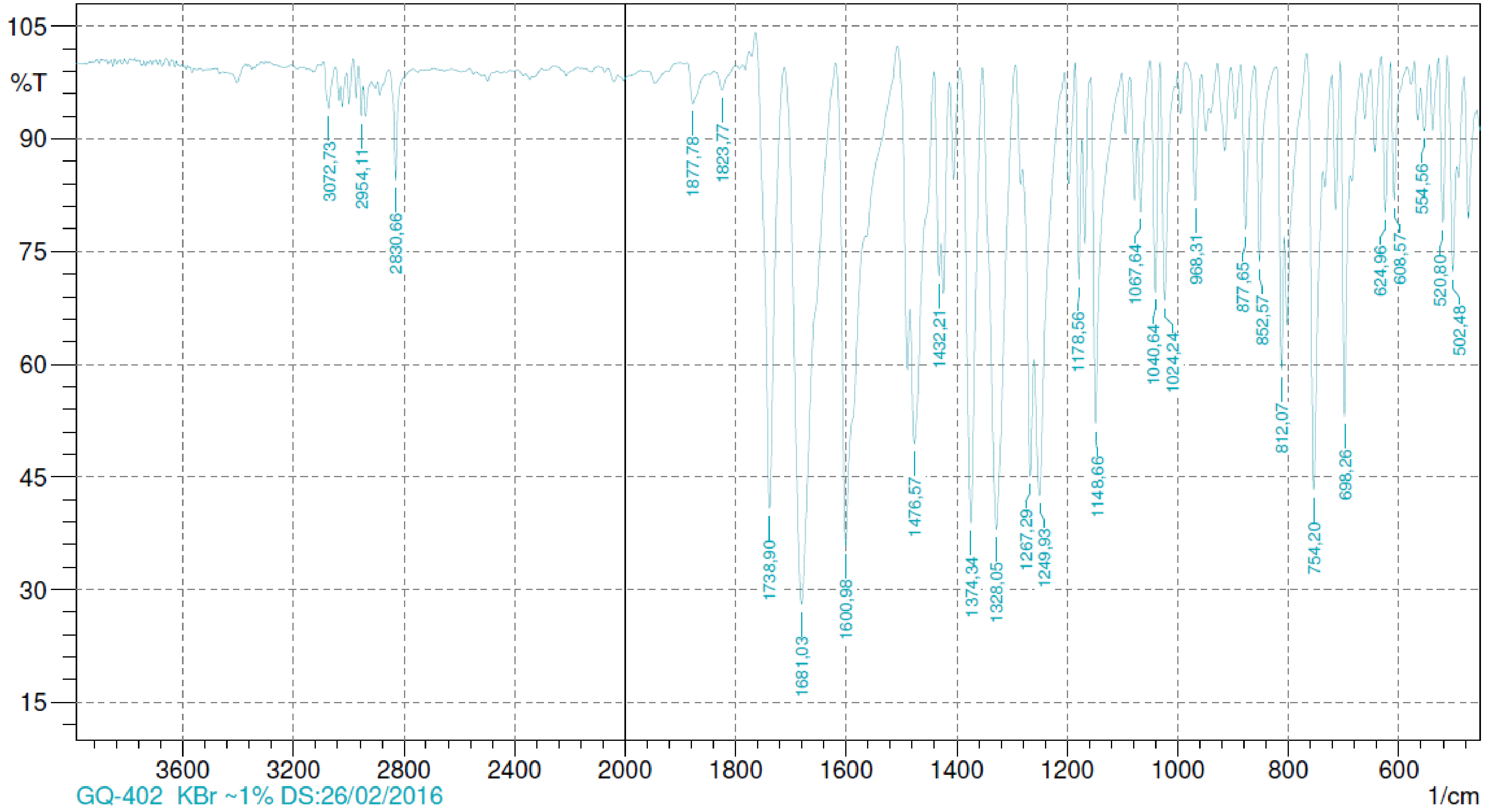

Comment;
GQ-402 KBr ~1% DS:26/02/2016

Date/Time; 25/5/2016 14:15:45

No. of Scans;

Resolution;

Apodization;

User; Marina Pitta

MS

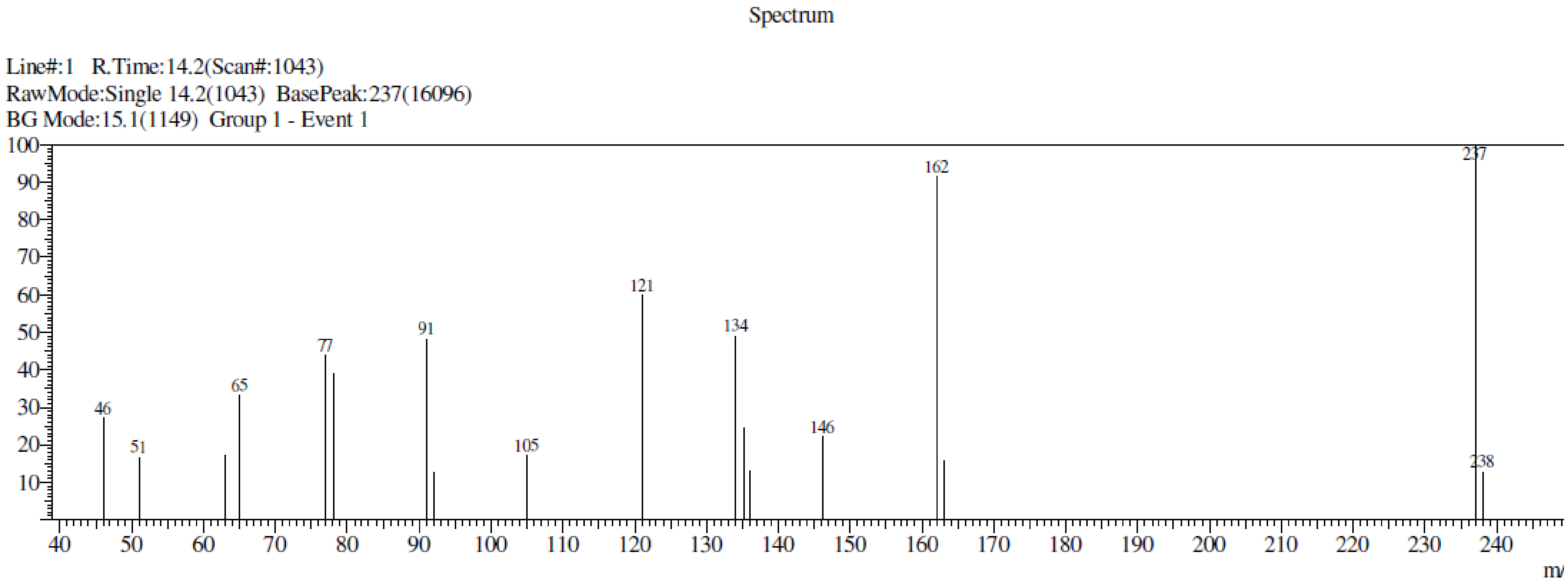
Spectrum
Line#:1 R.Time:14.2(Scan#:1043)
RawMode:Single 14.2(1043) BasePeak:237(16096)
BG Mode:15.1(1149) Group 1 - Event 1
100
90
80
70
60
50
40
30
20
10
46
51
65
77
91
105
121
134
146
162
237
238
40
50
60
70
80
90
100
110
120
130
140
150
160
170
180
190
200
210
220
230
240
m/

GQ-396

Chromatogram
GQ-396 C:\LabSolutions\Data\GQ-396.lcd

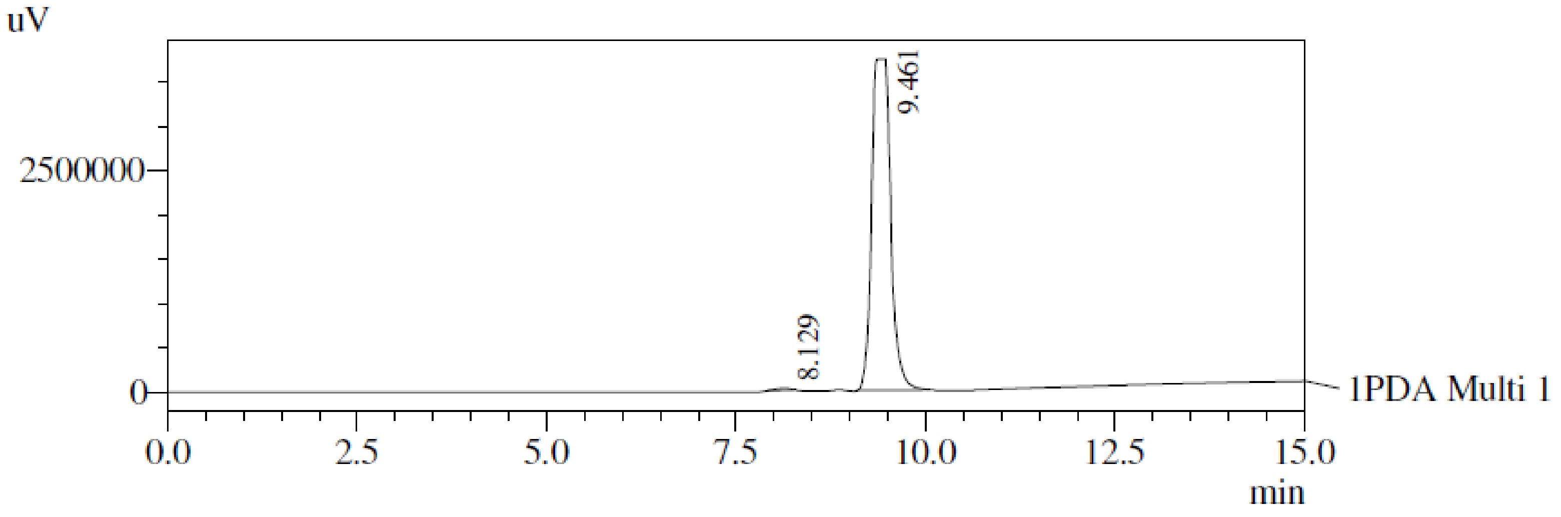

1 PDA Multi 1 / 205nm 4nm

<Target Spectrum>
ID# : 1
Retention Time : 8.129
Compound Name : IP-51
Spectrum Operation : None

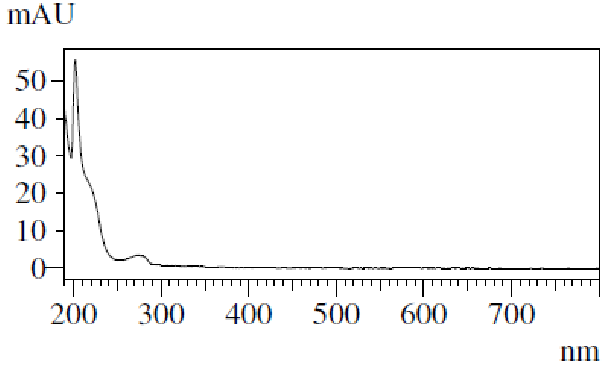

PeakTable

PDA Ch1 205nm 4nm

Peak#	Ret. Time	Area	Height	Area %	Height %
1	8.129	298881	20911	0.459	0.557
2	9.461	64799273	3734317	99.541	99.443
Total		65098153	3755228	100.000	100.000

GQ-396 1H NMR

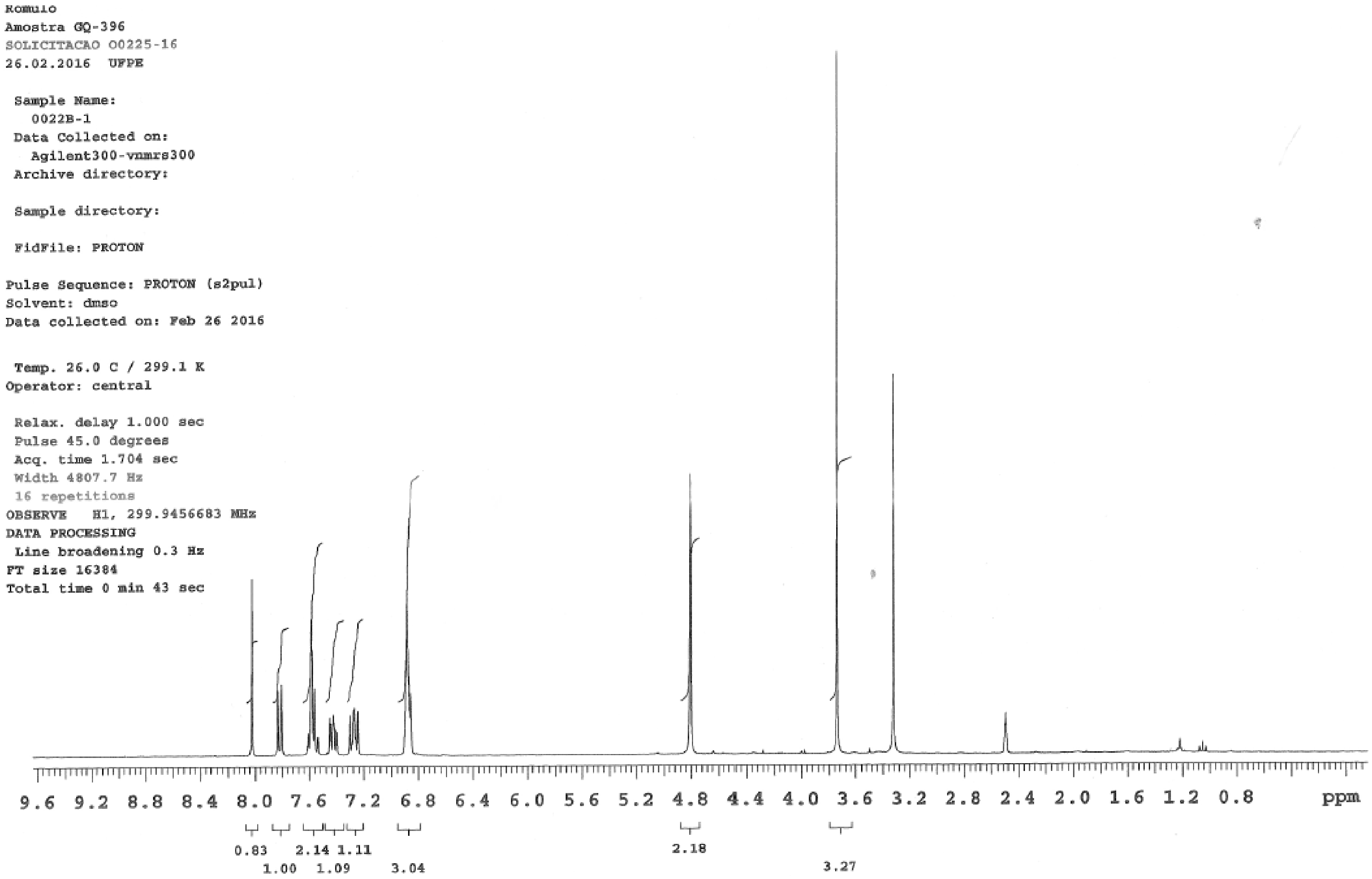
Romulo
Amostra GQ-396
SOLICITACAO 00225-16
26.02.2016 UFPE
Sample Name:
0022B-1
Data Collected on:
Agilent300-vnmrs300
Archive directory:
Sample directory:
FidFile: PROTON
Pulse Sequence: PROTON (s2pul)
Solvent: dmso
Data collected on: Feb 26 2016
Temp. 26.0 C / 299.1 K
Operator: central
Relax. delay 1.000 sec
Pulse 45.0 degrees
Acq. time 1.704 sec
Width 4807.7 Hz
16 repetitions
OBSERVE H1, 299.9456683 MHz
DATA PROCESSING
Line broadening 0.3 Hz
FT size 16384
Total time 0 min 43 sec
9.6 9.2 8.8 8.4 8.0 7.6 7.2 6.8 6.4 6.0 5.6 5.2 4.8 4.4 4.0 3.6 3.2 2.8 2.4 2.0 1.6 1.2 0.8 ppm
0.83 1.00 2.14 1.09 1.11 3.04 2.18 3.27

GQ-396

13C NMR

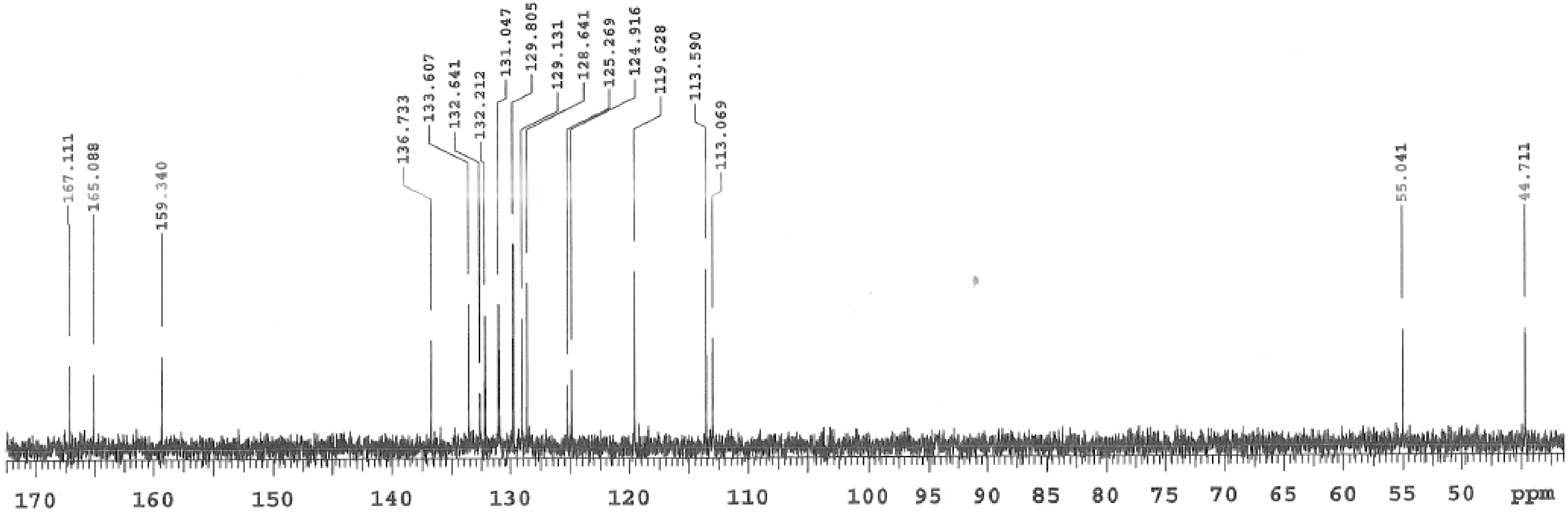

IV

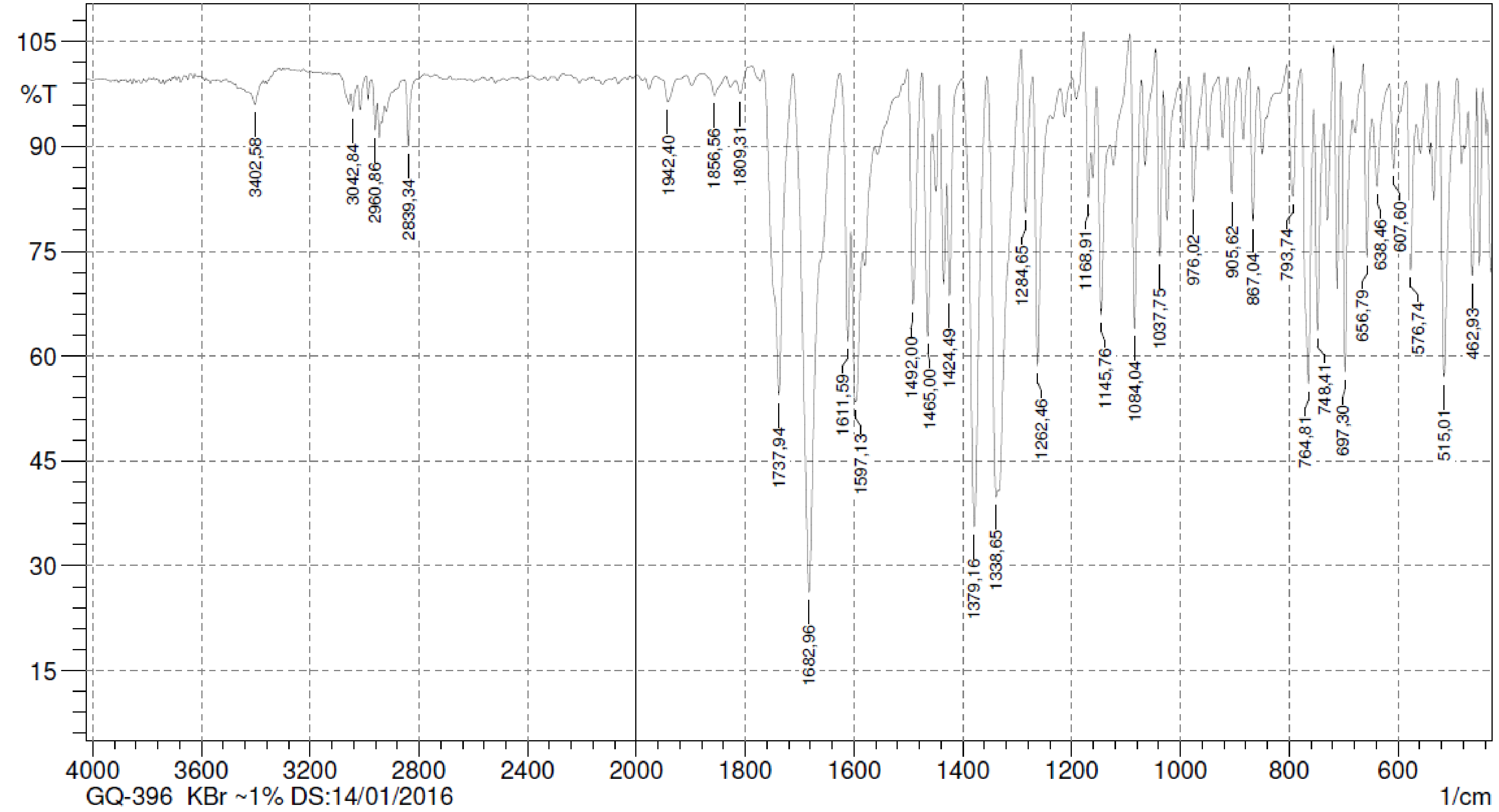

Comment;
GQ-396 KBr ~1% DS:14/01/2016

Date/Time; 26/7/2016 09:04:07

No. of Scans;

Resolution;

Apodization;

User; Marina Pitta

MS

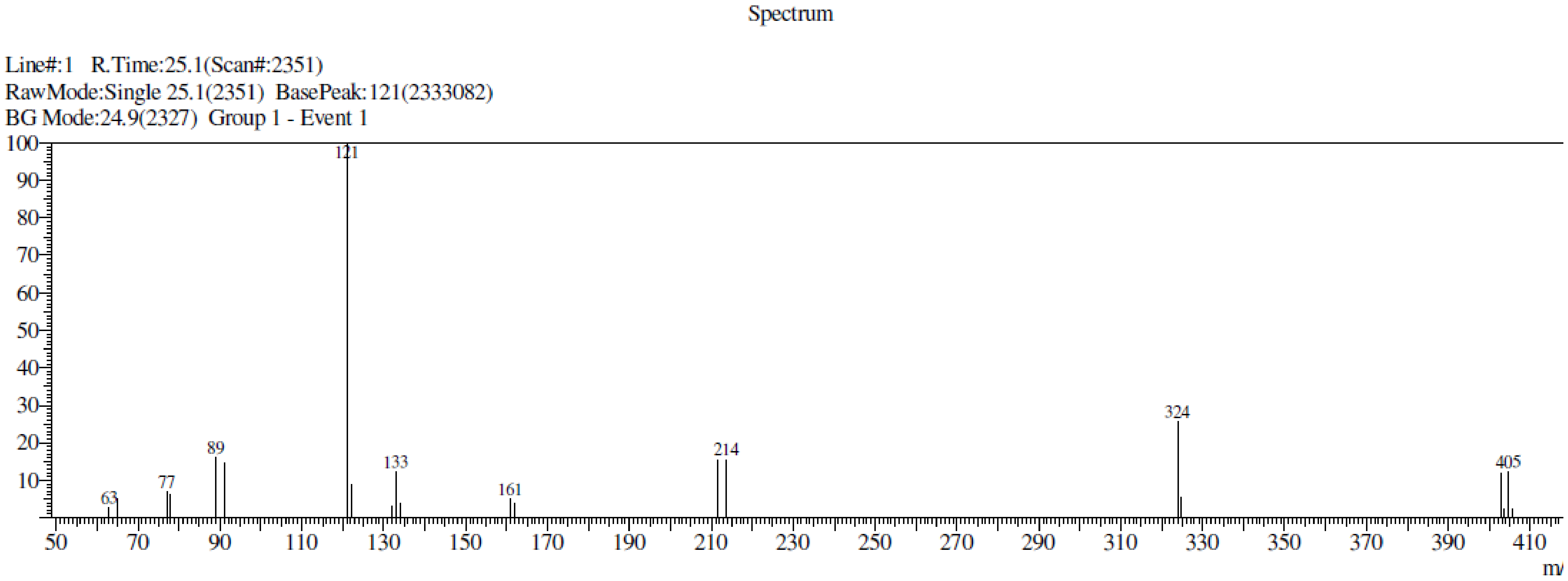
Spectrum
Line#:1 R.Time:25.1(Scan#:2351)
RawMode:Single 25.1(2351) BasePeak:121(2333082)
BG Mode:24.9(2327) Group 1 - Event 1
100
90
80
70
60
50
40
30
20
10
121
324
214
89
133
405
161
77
63
50
70
90
110
130
150
170
190
210
230
250
270
290
310
330
350
370
390
410
m/
